# Aggressive vascular tumor mimicking posttraumatic hematoma: A case report of kaposiform hemangioendothelioma on the nose

**DOI:** 10.1016/j.jdcr.2022.05.041

**Published:** 2022-06-09

**Authors:** Floris V.W.J. van Zijl, Peter C.J. de Laat, Robert M. Verdijk, Andries P. Nagtegaal, Frank R. Datema

**Affiliations:** a Department of Otorhinolaryngology and Head and Neck Surgery, Erasmus Medical Center, Rotterdam, the Netherlands; b Department of Paediatrics and Paediatric Vascular Anomaly Center (WEVAR), Erasmus Medical Center Sophia Children’s Hospital, Rotterdam, the Netherlands; c Department of Pathology, Erasmus Medical Center, Rotterdam, the Netherlands

**Keywords:** hemangioendothelioma, Kaposiform, nose, trauma, tumor, vascular, KHE, kaposiform hemangioendothelioma, KMP, Kasabach-Merritt phenomenon

## Introduction

Kaposiform hemangioendothelioma (KHE) is a rare, locally aggressive vascular tumor presenting in infancy or early childhood. Typically, KHE is located in the skin or subcutaneous soft tissue of the extremities, trunk, retroperitoneum, or neck.^1^ Here, we report a case of KHE localized on the nose that manifested after nasal trauma. The diagnostic delay resulting from this posttraumatic presentation, as well as the response to prednisolone and sirolimus with 5 years of follow-up are presented. This case emphasizes the clinical importance of screening for underlying pathology in children with persistent sequelae after nasal trauma.

## Case report

A 2-year-old Mediterranean boy was referred to our department for reevaluation of a persistent purpuric lesion of the nose. Six months earlier, the boy fell on his nose and developed an apparent hematoma on the nasal dorsum that was initially considered to be secondary to the nasal contusion. However, the lesion did not resolve in the following months and even expanded during heat exposure. His parents denied preexisting nasal abnormalities, supported by photographs taken a few days preceding the accident. The boy was otherwise healthy with no history of skin lesions.

**Fig 1:**
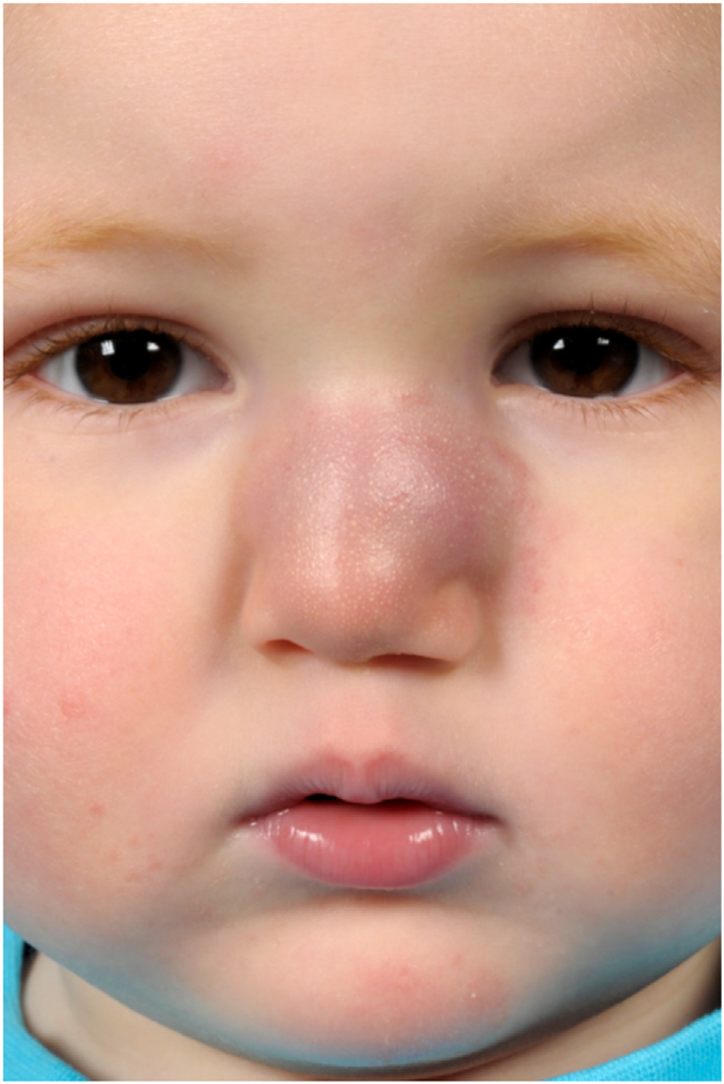
At presentation, 6 months after trauma. A red-purple plaque with ill-defined borders was located on the nasal dorsum with extension to the left paranasal area.

**Fig 2:**
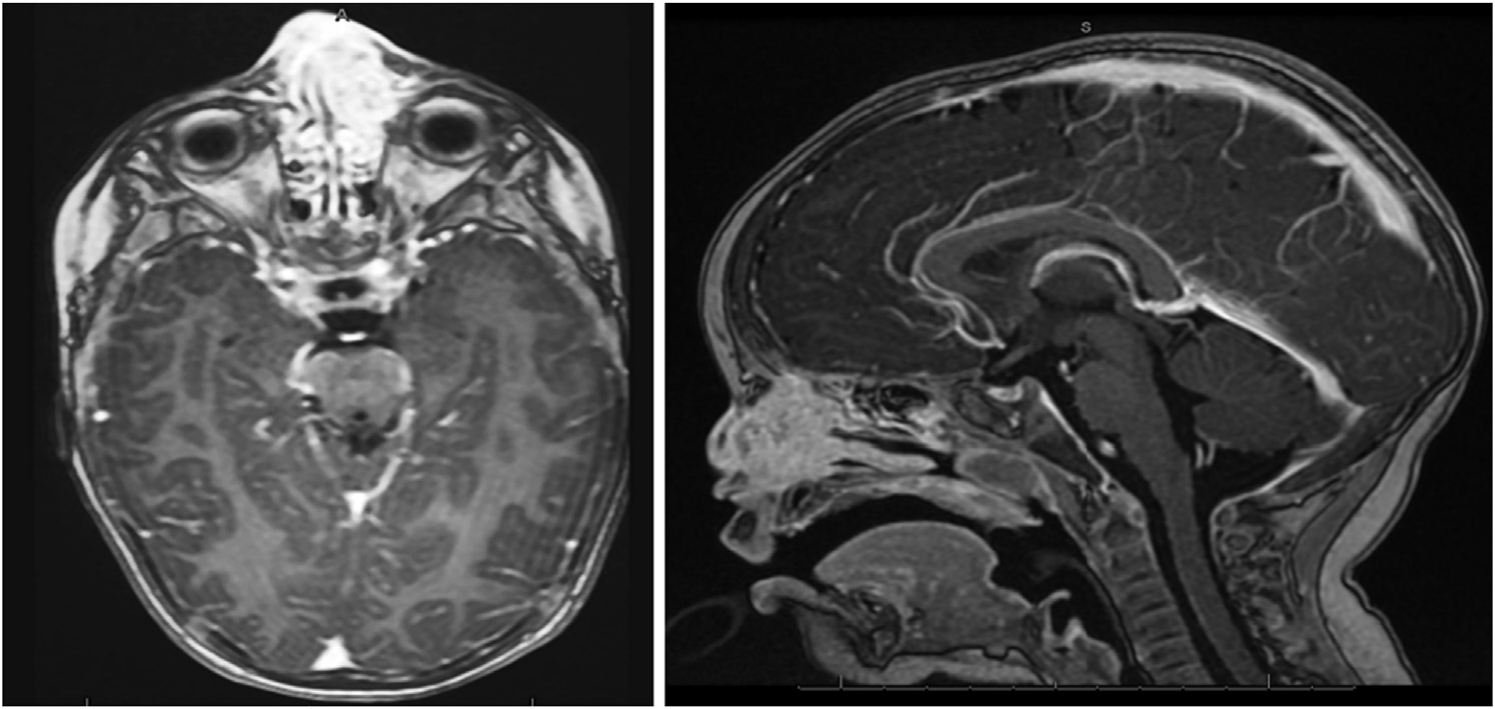
Axial T1-sequence with gadolinium, axial (*left*) and sagittal (*right*). Diffuse subcutaneous enhancement was seen, extending into the left anterior ethmoid.

**Fig 3:**
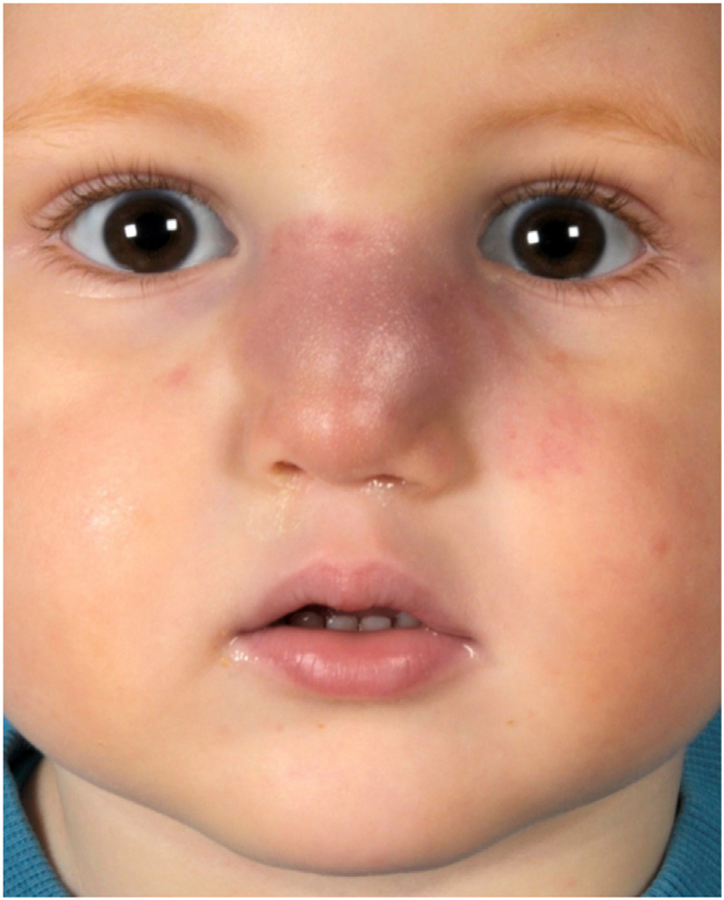
Enlargement of the lesion while on the waiting list for surgery.

**Fig 4:**
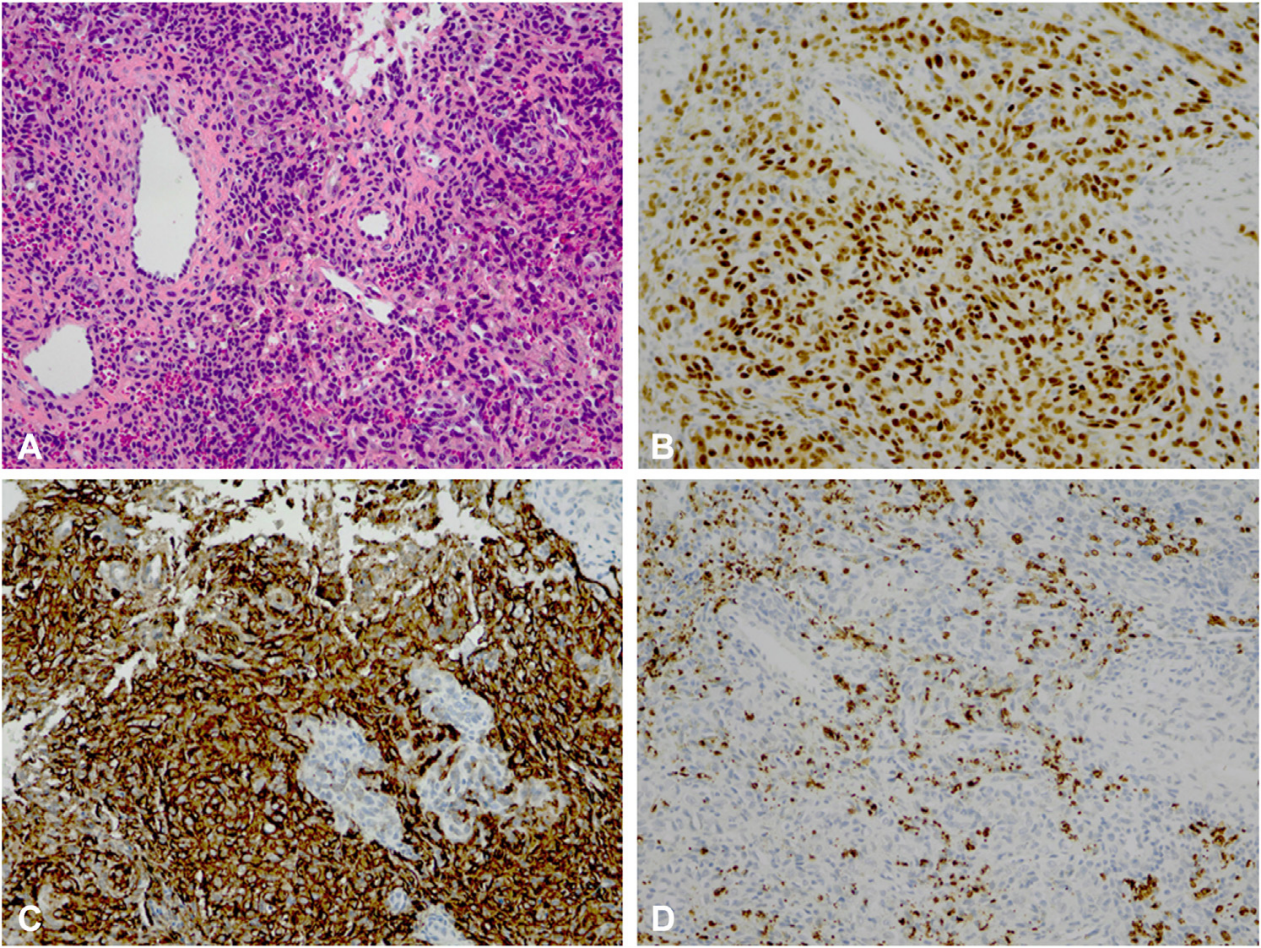
**A,** Histopathology showing infiltrating sheets of plump spindled endothelial cells. **B,** The endothelial cells showed nuclear positivity for ERG. **C,** The lesional cells demonstrated nuclear positivity for D2-40. **D,** Glut1 staining is negative in the tumor cells (erythrocytes are positive) excluding infantile hemangioma.(**A,** Hematoxylin-eosin stain; original magnification: ×200.)

Physical examination revealed a firm, red-purple swelling of the skin and subcutaneous tissues involving the nasal dorsum and paranasal area (Fig 1). Small pearls of clear fluid were visible on the affected skin. Congested mucosa and crusting were noted upon endonasal inspection. A magnetic resonance imaging scan revealed a subcutaneous infiltration extending into the nasal cavity and anterior ethmoid sinus with bright heterogeneous enhancement on T1 with gadolinium (Fig 2). No connections between the lesion and intracranial dural sinuses were seen, ruling out sinus pericranii. A Doppler ultrasound found rich vascularization without signs of lymphangioma or traumatic arteriovenous malformation. Since trauma was considered the etiologic factor, an infected, organized hematoma was suspected. The patient was started on amoxicillin/clavulanic acid for two weeks without any response. A surgical exploration to rule out underlying pathologies such as a foreign body, dermoid cyst, or lymphatic malformation was scheduled. While on the waiting list for surgery, the boy presented with a sudden enlargement of the lesion and episodic epistaxis without signs of infection (Fig 3). To rule out malignancy, the deformity was immediately biopsied using an endonasal incision under general anesthesia. Histopathology revealed infiltrating sheets of plump spindled endothelial cells that were ERG and D2-40 positive and negative for Glut1 and HHV8. These findings are consistent with KHE (Fig 4).

**Fig 5:**
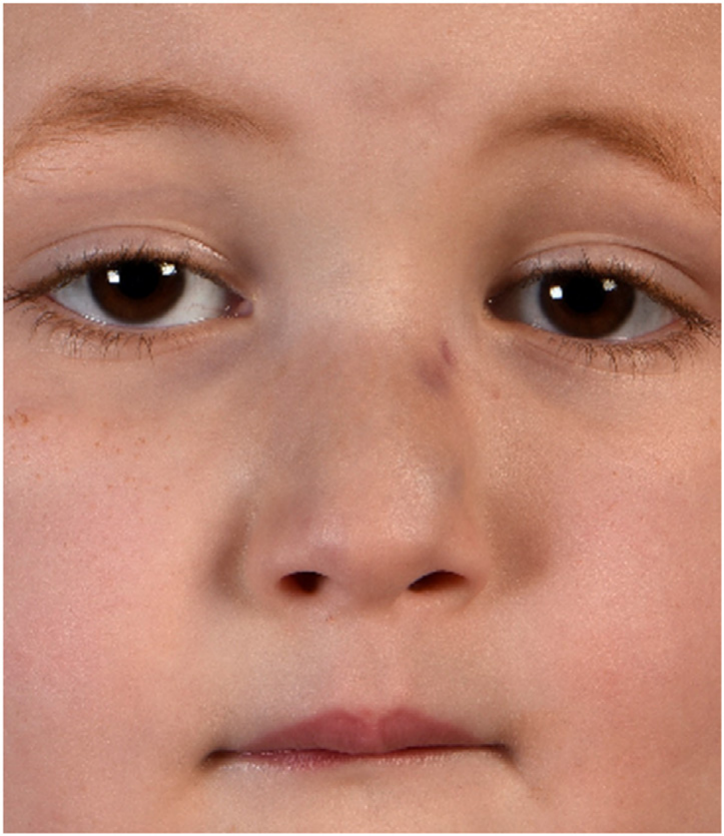
Result after 5 years of treatment with prednisolone and sirolimus.

Due to the size and location, the tumor was considered inoperable, and the Pediatric Vascular Anomaly Team was consulted. The patient did not respond to initial treatment with atenolol 1 mg/kg. Subsequent treatment with prednisolone 2 mg/kg effectuated only limited resolution and resulted in severe Cushing syndrome and growth retardation. Moreover, flare-ups of the lesion were noted during tapering. One year after presentation the patient was started on sirolimus 1.8 mg/m^2^/day, resulting in a gradual regression of the tumor on both physical examination and magnetic resonance imaging. After 4 years of treatment with sirolimus, the patient demonstrated marked clinical improvement (Fig 5).

## Discussion

For most dermatologists, otolaryngologists, and pediatricians, KHE is an uncommon disease not likely to be part of the differential diagnosis of a red-purple subcutaneous nasal lesion. Although no epidemiologic studies have reported the incidence or prevalence of KHE, it is considered a very rare entity. The diagnosis of this predominantly pediatric tumor is based on a combination of clinical, radiologic, and histologic features. Typically, magnetic resonance imaging demonstrates an ill-defined soft tissue mass, hypo- or isointense on T1-weighted sequences, and hyperintense on T2-weighted sequences, with enhancement post-gadolinium.^2^ Histologically, the tumor displays infiltrating nodules and sheets of spindled endothelial cells reminiscent of Kaposi sarcoma, hence the term “kaposiform.” Immunohistochemical properties of KHE can differentiate these tumors from infantile hemangiomas, Kaposi sarcoma, and lymphatic malformations.^3^ In patients with KHE, laboratory tests are required to rule out the Kasabach-Merritt phenomenon (KMP), a potentially life-threatening complication almost exclusively associated with KHE. KMP is characterized by thrombocytopenia and consumption coagulopathy and develops in approximately 60% to 70% of KHE cases, particularly those with larger tumors.^1^^,^^4^ In our patient, with a relatively small lesion, KMP was absent.

To our knowledge, there are no reported cases of KHE located in the skin-soft-tissue envelope of the nose. Following a history of nasal trauma, this localization is specifically deceitful as the clinical presentation of KHE is analogous to posttraumatic nasal hematoma. An etiologic relationship between trauma and KHE is unlikely; almost all KHE cases arise without any obvious cause.^5^ Yet, several reports suggest a link between local trauma, biopsy, or infection.^6^, ^7^, ^8^ Others report a link between trauma or vaccination and the development of KMP in patients with KHE.^9^^,^^10^ These findings raise the suggestion that trauma and inflammation contribute to the aggravation of KHE. We hypothesize that in our case, the trauma provoked an intralesional hemorrhage which manifested as a dramatic enlargement of the previously subclinical KHE. Therefore, in patients with persistent sequelae after trauma, KHE should be included in the differential diagnosis.

Although KHE may spontaneously decrease in size with time, complete regression is uncommon. Surgical excision is the treatment of choice in small, localized lesions, but the infiltrative nature of KHE often precludes complete resection. Moreover, surgery in neonates and young patients poses significant mortality and morbidity, the latter especially for lesions on the face. Medical management of KHE traditionally consists of corticosteroids or vincristine, with varying response rates. Over the last decade, an increasing number of studies have demonstrated promising responses to sirolimus, an inhibitor of the mammalian target of rapamycin.^5^ Presumably, its mechanism of action is the inhibition of cell proliferation and metabolism, lymphangiogenesis, and angiogenesis. Long-term data concerning response rates and side effects are lacking, but sirolimus appears to be well-tolerated and efficacious in the short term and can be administered orally, as opposed to vincristine. Our case underlines the success of sirolimus in the treatment of KHE, although resolution may take several years.

## Conflicts of interest

None disclosed.
